# Free Trade in Medicine

**Published:** 1890-09-06

**Authors:** 


### The Hospital

A Weekly Journal of

Science, Medicine, Nursing, and Philanthropy.

*For Hospitals, Asylums, Medical Practitioners, Students, Nurses, Families, Parishes, Congregations, and the Charitable Public.*

Vol. VIII.—No. 206.] September 6, 1890.

### Free Trade in Medicine.

Sir George Grey, it is said, is going to bring before the New Zealand Legislature a measure for the throwing open of the medical profession. What is meant, we suppose, is that everybody in the Colony shall be enabled to practice the art of medicine for money, whether he be legally qualified and registered or not. The question is one which belongs to the public entirely. The Legislature either of New Zealand or of Great Britain has a perfect right to take such a step as Sir George Grey intends to propose; and if statesmen are convinced that the throwing open of the medical profession will serve the public interests, it is their duty to throw it open. Medical men, who are also intelligent politicians, will see no professional reason why an experiment of the kind should not be made, particularly if it is to be made on a small scale. They feel quite confident that they can hold their own against the quack and the pretender exactly as the properly-trained tradesman can live and gain wealth in spite of the rival amateur. There is little doubt indeed that the work of skilled practitioners would be increased if an army of ignorant quacks were let loose upon a too confiding public.

But, in spite of these academic opinions, we are about to try to show cause why the practice of medicine should not be thrown open to all and sundry. If any too clever critic sees the cloven hoof here, he is welcome to the enjoyment of his discovery. For our part, we look upon the medical profession not merely as a number of individual practitioners working for bread and butter, but as the proper and natural custodians of the public health. Is there, or is there not, a department of public health in this country? There is such a department; and it owes its existence entirely to doctors. Is there, or is there not, in the present day, an enthusiastic crusade against all insanitary and life-endangering ways? And who are the crusaders? They are doctors almost to a man! Medical men know full well that the prevalence of disease is their harvest time, their period of brisk and prosperous trade; and yet they never cease to raise their voices in the most strenuous advocacy of sanitation and wholesome living. Thus does their humanity triumph at the expense of their material prosperity. We are not claiming for doctors that they are more humane by nature than other men. Their work is such that larger claims are made upon their public and private humanity than is the case with other men; and, in responding to those claims, they only do what all other well-conditioned persons would do in the like circumstances.

Free trade in medicine looks as if it would make as good a "cry" as a free breakfast-table. But the country has had experience of a medical profession controlled and limited by parliamentary enactments, and of a medical profession free and open to the whole world; and the experience has been sufficiently long in each case. Every intelligent reader should ask whether the "free" or the "state controlled" doctors have proved the most capable practitioners of their art? And whether the health of the public and the annual death-rate have been more satisfactory under the "free" or under the "controlled" régime? Of course this is an appeal to the self-interest of the public. But the very highest morality would not ask of the public that it should sacrifice its solid and proved interests in order that it may give freedom to all sorts of persons to practise an art which they do not understand. The State ought to give all possible liberty to individuals; but it should give nobody the liberty to steal or to kill. Neither should it give any untrained person the liberty to tamper with limb or life solely for his own gains.

Before the year 1815 there was no State Control over medical men. Any person who chose to do so could practise medicine without let or hindrance. The result was that a great many persons did practise without any kind of legal qualification, and had done so from the earliest times. But what was the condition of the medical profession in this country as a whole before 1815? Nothing that we could say would convey to the general reader an adequate presentment of the actual state of things; because the general reader does not know anything scientifically about physiology, disease, and the use of drugs; and, therefore, he has no standard whereby to estimate the degree of ignorance of untrained as compared with trained practitioners, or the risks that men run when they entrust such an organism as the human body to the tinkering manipulation of those who know nothing about it. But this much is matter of history, that less than a century ago the science of physiology and the almost equally important science of morbid anatomy did not exist. The most eminent physicians, as well as the most incompetent, were empirics pure and simple. Now nobody, except a grossly ignorant person, will speak against those who practise an art empirically when they have no means of practising it in any other way. Illiterate doctors and other uneducated persons have frequently denounced empiricism without apparently having the least idea what empiricism is. An empiric is simply a man who uses the best knowledge he has and the best methods he knows for the purposes of his business. Most farmers are empirics; but they manage to raise corn and cattle enough to feed thirteen hundred millions of people every year. We do not then undervalue empiricism. But nobody, surely, will compare the empiric who does his best to correct the vagaries of a complex organism which he knows little or nothing about with the scientific practitioner who knows every detail of the minute structure of the organism, and every mode of its functional activity! The physicians of pre-scientific days, when they were competent and honest, were able, by diligent watchfulness, to accumulate large stores of priceless experience; and with such experience in their minds and fingers they managed their cases with wonderful skill and judgment, and often with conspicuous success. But such physicians were the rare exceptions. The majority of the profession were not merely destitute of scientific knowledge, but also of practical skill. Many of them were the most ignorant of pretenders, and they knew they were so, and, therefore, they added hypocrisy and knavery to their other accomplishments. It was not Bacon alone who poured contempt upon the medical practitioners of his time; the whole country held them in derision, and in many cases in undisguised aversion. Not even to-day have medicine and surgery fully recovered from the degradation into which they were plunged by our professional ancestors.

So far as the public of some earlier centuries were concerned they would often have been a great deal better without any treatment at all than with such as they were able to procure. Hecker, in his "Epidemics of the middle ages," gives an account of the "Sweating Sickness" which visited England in the fifteenth century. He says: "The physicians could do little or nothing for the people in their extremity. . . . This holds good even of the famous Thomas Linacre, subsequently physician in ordinary to two Monarchs, and founder of the College of Physicians. . . . In fact the restorers of the medical science of ancient Greece, who were followed by all the most enlightened men in Europe, with the single exception of Linacre, occupied themselves rather with the ancient terms of art, than with actual observation. . . . This reminds us of the later Greek physicians, who for 400 years paid no attention to the small-pox, because they could find no description of it in the immortal works of Galen. . . . No resource was therefore left to the terrified people of England but their own good sense, and this led them to the adoption of a plan of treatment, than which no physician in the world could have given them a better." There is no doubt that Hecker, himself a physician, gives here a perfectly fair description of the sort of medical practice that prevailed for hundreds of years during which there was free trade in medicine.

Is it necessary, by way of contrast, to speak at any length of the scientific knowledge and skill of the medicine and surgery of to-day? Medicine, as a science, may indeed almost be said to date its birth from the time when a moderately efficient training was insisted upon by the State. The improvement that was initiated then, has been constantly growing up to the present time, and promises to grow in an ever increasing ratio. It is quite certain that the obscurest village practitioner of our own day knows more of physiology and of disease and its treatment than the most eminent Court physicians of the Tudors and the Stuarts. That all this is due to the State control which insists upon the adequate education and examination of medical men we do not affirm. But it is, at least, a striking circumstance that medicine and surgery should have progressed by leaps and bounds under State control, whilst in the old days their utter stagnation should have been a disgrace both to medical men and the country. Of the advantage to the public of the new state of things there is neither space nor necessity to speak.

